# Hepatoprotective Properties of *Opuntia ficus indica* Cladodes Against NiSO_4_‐Induced Oxidative Stress and Liver Damage

**DOI:** 10.1002/fsn3.71044

**Published:** 2025-10-02

**Authors:** Sara Razzak, Marouane Aouji, Soufiane El Assri, Chaima Sabri, Abdessamad Ittorahou, Laila Ibouzinedine, Amir Bouallegue, Esmael M. Alyami, Abdulrahman A. Almehizia, Farid Khalouki, Fakhreldeen Dabiellil, Mohammed Bourhia, Anass Haloui, Youness Taboz

**Affiliations:** ^1^ Natural Resources and Sustainable Development Laboratory, Research Unit in Nutrition, Metabolism and Physiology, Department of Biology, Faculty of Sciences Ibn Tofail University Kenitra Morocco; ^2^ Biochemistry Laboratory, Central Laboratory Service Mohammed VI University Hospital, Faculty of Medicine and Pharmacy of Oujda Mohammed First University Oujda Morocco; ^3^ Biology and Health Laboratory, Faculty of Sciences Ibn Tofail University Kenitra Morocco; ^4^ Laboratory of Food Oral Processing, School of Food Science and Biotechnology Zhejiang Gongshang University Hangzhou China; ^5^ Department of Biology, College of Science King Khalid University Abha Saudi Arabia; ^6^ Department of Pharmaceutical Chemistry, College of Pharmacy King Saud University Riyadh Saudi Arabia; ^7^ Biology Department, FSTE Moulay Ismail University of Meknes Errachidia Morocco; ^8^ University of Bahr el Ghazal Wau South Sudan; ^9^ Laboratory of Biotechnology and Natural Resources Valorization, Faculty of Sciences Ibn Zohr University Agadir Morocco; ^10^ Laboratory of Pathological Anatomy, Mohammed VI University Hospital, Faculty of Medicine and Pharmacy of Oujda Mohammed First University Oujda Morocco

**Keywords:** cladode juice, hepatic biomarkers, hepatoprotective, histological analysis, molecular docking, NiSO_4_, oxidative stress

## Abstract

The *Opuntia* genus exhibits several mechanisms that support liver function. Due to its richness in phenolic compounds, our study highlights the hepatoprotective potential of *Opuntia ficus‐indica* cladode juice in mitigating nickel‐induced liver injury. The polyphenol content was determined using spectrophotometric analysis and HPLC‐MS. Experimental hepatotoxicity was induced by intraperitoneal (i.p.) injection of NiSO_4_. To assess the hepatoprotective effects of cladode juice, various analyses were performed to evaluate its impact on enzymatic activity, serum biomarkers, oxidative stress markers, and liver tissue structure. Quantification of phenolic compounds confirmed the presence of total polyphenols and flavonoids. Chromatographic analysis identified p‐coumaric acid, piscidic acid, isorhamnetin‐3‐O‐neohesperoside, and ellagic acid as the major phenolic constituents. Cladode juice exhibited a hepatoprotective effect in rats intoxicated with nickel sulfate (NiSO_4_), as evidenced by the modulation of plasma hepatic biomarkers, restoration of oxidative stress marker levels, and a marked reduction in hepatic lesions observed through histological analysis. Molecular docking studies of the four identified polyphenolic compounds with alanine transaminase revealed a higher binding affinity for ellagic acid, followed by p‐coumaric acid. These beneficial effects are attributed to the plant's diverse bioactive compounds, highlighting their potential to mitigate oxidative damage and support liver function.

## Graphical abstract

Our study suggests that *Opuntia ficus indica* cladode juice exhibits hepatoprotective properties, mitigating liver damage induced by nickel exposure. The polyphenol content was determined using spectrophotometric analysis and HPLC‐MS. Experimental hepatotoxicity was induced by intraperitoneal (i.p.) injection of NiSO_4_.

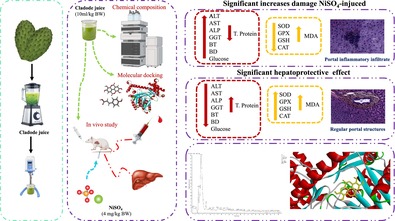

## 1 Introduction

Heavy metal pollution is a global problem that threatens human and animal health and also the environment. Human activities, including industry, agriculture, and transport, release heavy metals into the air, water, and soil (Brown and Margolis 2012). Unlike heavy metals, there are light metals such as nickel (Ni), which can become toxic if present at high levels (Iyaka 2011). The 24th most prevalent element in the crust of the planet is nickel (Ni), accounting for 3% of its composition, second only to iron, oxygen, magnesium, and silicon. Nickel metals and their alloys are widely used in the food industry, particularly as catalysts and pigments, in chemical and metallurgical applications. Nickel is recognized as a valuable micronutrient for specific plant species (Liu et al. 2011) and a crucial trace element for the human body (Cao et al. 2016); nonetheless, excessive concentrations can have adverse effects. The exposure to high levels of nickel has been associated with the development of cancer and various health complications such as fatigue, headaches, dermatitis, vertigo, cardiovascular issues, and respiratory disorders (Pasha et al. 2010). Commercially important nickel salts include nickel chloride, nickel sulphate, nickel nitrate, nickel carbonate, nickel hydroxide, nickel acetate, and nickel oxide (Cempel and Nikel 2006). Nickel is a metal that can accumulate in the body, particularly in the bones, liver, and kidneys. This bioaccumulation can have negative health consequences, based on the dosage and duration of contact. Nickel is an immunotoxic and carcinogenic agent that can damage the immune system and cause disease (Genchi et al. 2020). However, excessive consumption may lead to oxidative stress, resulting in an overproduction of harmful free radicals. This can damage cellular structures and impair liver function, potentially resulting in severe liver toxicity (Meng et al. 2018).

The liver is the organ most sensitive to nickel toxicity, whether of environmental or occupational origin. Because nickel disrupts the metabolism of ascorbate‐cholesterol and changes the activity of various enzymes, it can cause significant damage to the liver and kidneys (Derbal et al. 2022). The production of reactive oxygen species (ROS) and a rise in lipid peroxidation (LPO) at the cellular level are two negative consequences of nickel's action in the body. Nickel accumulation is responsible for increased production of ROS and increased LPO (Guo et al. 2019). The exposure to nickel has been correlated with alterations in the functionality of various antioxidant enzymes, which encompass catalase (CAT), reduced glutathione (GSH), glutathione peroxidase (GPX), and glutathione S‐transferase (GST). These enzymes assume a critical function in the detoxification of reactive oxygen species (ROS) and the safeguarding of cellular integrity against damage (Sidhu et al. 2004). Moreover, oxidative stress, a condition arising from a lack of balance in the middle of antioxidant defenses and reactive oxygen species, is implicated throughout the initiation and development of liver damage (Li et al. 2015). Due to its significant role in liver injury, antioxidants have been thoroughly investigated for their possible help in treating and preventing liver disorders, both acute and chronic (Adak 2018). For this reason, numerous studies have demonstrated the positive effects of antioxidants, especially in the prevention and treatment of liver injury. Aggressive antioxidant therapy is thought to enhance liver injury outcomes. Furthermore, using synthetic medications to treat liver illness may not be effective or may result in major side effects (Touiss et al. 2021).

The hepatoprotective potential pertains to the ability of specific compounds to mitigate hepatic injury, safeguarding this vital organ engaged in numerous biochemical pathways. Multiple investigations have substantiated the efficacy of various agents in preserving liver integrity (Sharukh et al. 2023).

The genus *Opuntia* provides a variety of mechanisms and benefits for liver function. Antioxidant action is a notable mechanism exhibited by several species within this genus (Chen et al. 2021). The liver‐protective properties of cactus cladodes, particularly those from *Opuntia atropes* and *Opuntia ficus indica*, are linked to their abundant phytochemical profile, including flavonoids and dietary fibers, which exhibit notable antioxidative potential. Investigations indicate that cactus cladode extracts can mitigate hepatic damage induced by toxins such as lithium carbonate and high‐fat diets, leading to reductions in hepatic lipid and triacylglycerol content by up to 73.1% in models used in experiments (Ben Saad et al. 2016; Duarte‐Medina et al. 2024). *Opuntia ficus indica* (*OFI*), a cactus of the Cactaceae family, has fruits and cladodes that are rich in essential nutrients, including minerals, vitamins, and antioxidants. Traditional medicine uses it to treat illnesses through these health benefits. The researchers have confirmed their efficiency in the treatment of multiple diseases thanks to these anti‐ulcerogenic, antioxidant, anti‐cancer, and hepatoprotective properties (Brahmi et al. 2011). Cladode juice's phenolic components, namely certain flavonoids and proanthocyanidins, along with beta‐carotene (provitamin A), vitamins C and E, and glutathione, have been linked to a reduction in blood glucose and cholesterol levels when consumed regularly (Hfaiedh et al. 2008). Furthermore, recent studies have substantiated the elevated concentration of some substances that are bioactive in cladodes, including phenolic compounds, phytosterols, betalains, carotenoids, and essential fatty acids, particularly linoleic and palmitic acids. This biochemical composition underscores their potential therapeutic and nutritional significance (Razzak, Aouji, Sabri, et al. 2024). Additionally, their rich nutritional profile, encompassing polysaccharides, vitamins, and minerals, further highlights their value in health and disease prevention (Razzak, Aouji, Zirari, et al. 2024).

Advanced computer‐assisted drug discovery technologies have advanced to allow the detection of drugs derived from phytochemical compounds present in various medicinal plants. In computer science, predictive models are essential for directing the methodological selection of technical and medical research. Additionally, they have been applied to the in silico prediction of toxicological, pharmacokinetic, and pharmacological performance (Loza‐Mejía et al. 2018). Currently, molecular docking represents an efficient and economical approach for the development and evaluation of pharmaceutical products. By using this technique, information on how pharmaceuticals interact with receptors is generated, which can be used to predict how drug candidates would align with their target protein (Lee and Kim 2019). In addition, this method promotes systematic exploration by positioning a molecule non‐covalently on the docking site of a target macromolecule, which leads to a specific association with the active sites of each ligand (Bharathi et al. 2014).

The main objective of this study was to evaluate the hepatoprotective capacity of phenolic compounds in cladode juice in laboratory animals. Using a rat model of NiSO_4_‐induced acute liver injury, we investigated the therapeutic properties of cladode juice extract from *Opuntia ficus indica* (*OFI*) and explored the mechanisms underlying its hepatoprotective efficacy. A molecular docking study involving alanine aminotransferase was also carried out to deepen the understanding of the mode of action of these molecules.

## 2 Materials and Methods

### 2.1 Cladodes Juice Preparation

Mature cladodes of the *Opuntia ficus indica* species were harvested in the commune of Oulad Boubker (Morocco). After the spines had been cleaned, the cladodes were cut into small pieces and mixed with a volume of distilled water (50%) using a Moulinex. The juice obtained was then filtered to remove the hard fibers and homogenized using a high‐speed blender (CAT Unidrive 1000 D).

### 2.2 Analysis of Phenolic Compounds

#### 2.2.1 Determination of Total Phenolic Content (TPC)

The Folin–Ciocalteu method, as described by Singleton et al. (1999), was used to determine the phenolic compound content in cladode extracts. Specifically, 0.5 mL of the sample extract was mixed with 2.5 mL of Folin–Ciocalteu reagent, which had been previously diluted tenfold with distilled water. Upon interaction with phenolic compounds, this reagent produces a blue coloration, the intensity of which is directly proportional to the phenolic concentration. After an 8‐min incubation period, 2 mL of a 7.5% sodium bicarbonate solution was added, followed by a 15‐min incubation in the absence of light. The absorbance was then measured at 765 nm, and the total phenolic content was quantified. Micrograms of gallic acid per milliliter of juice (mg GAE mL^−1^) were used to express the results. Three duplicates of the experiment were conducted.

#### 2.2.2 Determination of Total Flavonoid Content (TFC)

The total flavonoid content of cladodes was determined by colorimetric analysis following the protocol described by Djeridane et al. (2006). A reaction mixture consisting of 1 mL of extract and 1 mL of a 2% methanolic solution of aluminum chloride (AlCl_3_) was incubated for 15 min at room temperature. The absorbance was then measured using a spectrophotometer set at 430 nm. The reference blank for this analysis consisted of methanol combined with AlCl_3_. Flavonoid concentrations in the extracts were determined using a quercetin calibration curve, and the findings were reported as mg QE mL^−1^, or quercetin equivalent per milliliter of juice.

#### 2.2.3 Identification of Polyphenols by HPLC‐MS

Polyphenol Separation: A C18 column (5 μm, 250 × 4.6 mm) kept at 30°C was used to examine the material. Detection of UV/DAD was done between 200 and 600 nm. Phase A and Phase B mobile phases are 0.1% formic acid in water and 0.1% formic acid in acetonitrile, respectively. The injection volume was 10 μL, and the applied flow rate was 0.8 mL/min. 0–5 min at 15% A; 5–20 min at 15%–25% A; 20–30 min at 25%–35% A; 30–40 min at 35%–50% A; 40–50 min at 80% A; and 50–55 min at 15%. A was the gradient elution schedules. The following crucial characteristics were present in the system, which had a heated electrospray ionization (ESI) source and ran in either negative or positive ESI mode (±4.0 kV) (Shang et al. 2022).

– The flow rate of auxiliary gas is measured at 10 arbitrary units (arb) (Shang et al. 2022); the flow rate of sheath gas is quantified at 35 arb.
– The temperature of the capillary is maintained at 325°C; the temperature of the auxiliary gas heater is set at 350°C.
– The collision energies employed in a stepped and normalized fashion are quantified at 20, 40, and 60 eV (eV).
– The analytical procedure is conducted in full‐scan mass spectrometry mode with a resolution of 70,000 full width at half maximum (FWHM).
– The scanning range is established from m/z 10,0 to 100,000.

### 2.3 Animals

Twenty female albino Wistar rats with an initial weight of 205 ± 22 g were bred at the Faculty of Science, Ibn Tofail University, Kenitra 14000, Morocco. The rats were maintained on a 12‐h light/12‐h dark cycle and at a room temperature of 21°C ± 2°C. Access to water and food was free throughout the study. All experimental procedures were conducted following approval by the Institutional Ethics Committee for the Care and Use of Laboratory Animals at the Faculty of Sciences, ensuring full compliance with established animal welfare regulations.

### 2.4 Experimental Procedure

Rats were divided into four groups consisting of five animals in each group and treated for 40 days as follows: Control group: normal control rats, receiving distilled water (10 mL kg^−1^) daily for 30 days and injected with physiological saline (0.9% NaCl) at a dose of 4 mL kg^−1^ on days 31 to 40. Ni group: intoxicated control rats receiving distilled water (10 mL kg^−1^) daily for 30 days and injected with NiSO_4_ at a dose of 4 mL kg^−1^ on days 31 to 40. CJ group: rats receiving cladode juice (10 mL kg^−1^) daily for 30 days and injected with physiological saline (0.9% NaCl) at a dose of 4 mL kg^−1^ on days 31 to 40. CJ + NiSO_4_ group: rats receiving cladode juice (10 mL kg^−1^) daily for 30 days and injected with NiSO_4_ at a dose of 4 mL kg^−1^ on days 31 to 40. The study by Hfaiedh et al. (2008) was used as the basis for the nickel dose and experimental protocol in this study, with some modifications.

### 2.5 Serum Collection and Tissue Homogenate Preparation

Before each administration, the rats' body weight is recorded. After 24 h following the last NiSO_4_ injection, the rats are anesthetized with Pentobarbital. Heparin‐containing tubes are filled with blood drawn from the abdominal aorta. After centrifuging the samples for 10 min at 3000 rpm, the plasma is extracted and kept at −20°C for serum marker tests. After the liver is taken out, it is cleaned with physiological saline (NaCl, 0.9%), weighed, and 1 g of liver tissue is mixed with 5 mL of PBS (pH 7.4) in a Potter tube at 0 degrees Celsius (on ice). After that, the homogenate is centrifuged for 15 min at 14,500 rpm. The supernatant is stored at −20°C for oxidative stress assays. The remaining liver tissue is fixed in 10% formalin for histological analysis.

The following formula was used to calculate the liver index (Su et al. 2014):

$$\text{Liver index}\;\left(\%\right)=\left(\text{liver weight}/\text{body weight}\right)\times 100\%.$$

### 2.6 Analytical Methods

#### 2.6.1 Serum Marker Assays

All Serum marker enzyme experiments aspartate transaminase (AST), alanine transaminase (ALT), alkaline phosphatase (ALP), gamma glutamyl transferase (GGT), glucose, total protein, total and direct bilirubin (TB and DB) were conducted in triplicate for each specimen using the ROCHE COBAS INTEGRA 400 Plus autoanalyzer with the utilization of commercially available reagent kits.

#### 2.6.2 Estimate of Lipid Peroxidation (MDA)

By quantifying the production of malondialdehyde (MDA) in liver extracts, the level of lipid peroxidation (LPO) was indirectly assessed (Mihara and Uchiyama 1978). The concentration was expressed as nanomoles MDA/mg protein by measuring the absorbance of the complex formed at 535 nm.

#### 2.6.3 Estimation of Glutathione Reduction (GSH)

Hepatic glutathione (GSH) was measured by a colorimetric method known as the Ellman method. This method is based on the fact that glutathione reacts with 5,5′‐dithio‐bis(2‐nitrobenzoate) (DTNB) to form a yellow complex. The amount of GSH is proportional to the intensity of the yellow color (Ellman 1959). Expressed as nmol GSH/mg protein, it is the total GSH content of the sample.

#### 2.6.4 Glutathione Peroxidase Activity (GSH‐Px)

Glutathione peroxidase activity (GSH‐Px) was measured according to the method of Flohé and Günzler (1984). GSH‐Px activity was expressed as μmol GSH/min/mg protein.

#### 2.6.5 Catalase Activity (CAT)

The breakdown of hydrogen peroxide (H_2_O_2_) to water (H_2_O) and oxygen (O_2_) in the presence of the catalase enzyme at a wavelength of 240 nm was used to measure catalase activity. According to Aebi (1984), enzyme activity was measured as μmol H_2_O_2_ consumed/min/mg protein.

#### 2.6.6 Superoxide Dismutase Activity (SOD)

The inhibition of the reduction of nitro blue tetrazolium (NBT) is the principle of superoxide dismutase activity. Activity expressed in units/mg protein (Misra and Fridovich 1977).

### 2.7 Histopathological Study

The histopathological study was conducted at the Department of Pathological Anatomy, Mohammed VI University Hospital, Oujda. Organs harvested post‐mortem were weighed and preserved in a 10% formalin solution. Longitudinal sections were prepared using the paraffin embedding technique. Paraffin blocks were sectioned into 4–5 μm thick slices using a microtome (Leica RM2235) and stained with hematoxylin and eosin according to standard histological procedures. Microscopic observations and image capture were performed using a light microscope (Leica Microsystems) equipped with a camera (Leica DMD108) (Hewitson and Darby 2010).

### 2.8 Molecular Docking

As shown by the HPLC‐MS analysis, four molecules are present in the cladode of *Opuntia ficus indica* juice. The four ligands, namely ellagic acid, p‐coumaric acid, isorhamnetin 3‐o‐neohesperoside, and piscidic acid, were extracted from the PubChem database, while the biomacromolecule Alanine aminotransferase (PDB ID: 3IHJ) was obtained in PDB format. The ligands have been formatted as PDB files to simplify molecular research via PYMOL. In addition, Discovery Studio was used to prepare the proteins extracted from the PDB base. The active site of the enzyme was used to anchor the ligand structures thanks to AutoDockTools. The analysis of interactions between the active site residues of the enzyme and ligand molecules was conducted using Discovery Studio software, including a 2D interaction study (Zirari et al. 2025).

### 2.9 Statistical Analysis

Data are expressed as mean ± SEM. Statistical significance between groups was assessed using one‐way analysis of variance (ANOVA), followed by Tukey's post hoc test with a 95% confidence interval (*p* < 0.05), using SPSS software (version 27; IBM Corp., Armonk, NY, USA).

## 3 Results

### 3.1 Total Phenolic Concentration (TPC) and Total Flavonoid Concentration (TFC)

The assessment of bioactive constituents in *Opuntia ficus indica* cladode juice (CJ) extracted with different solvents was carried out using a colorimetric assay. The primary objective was to quantify phenolic compounds, as these organic molecules are largely responsible for the biological activities of the extract and contribute to mitigating oxidative damage induced by free radicals, which are implicated in various human pathologies. Phenolic compounds, abundantly distributed in the plant kingdom, exhibit noteworthy pharmacological properties (Kumari et al. 2017).

The findings from the quantification of total polyphenols and flavonoids revealed that all the cladode juices analyzed contained varying levels of these bioactive compounds.

The assessment of TPC using the Folin–Ciocalteu assay indicated that the average concentration in cladode juice, expressed in milligrams of gallic acid equivalent per milliliter, was 0.389 ± 0.04 mg GAE mL^−1^ (Table 1). This concentration reflects a significant presence of phenolic constituents, known for their potential involvement in various biochemical and protective mechanisms. In comparison with previous studies, Boutakiout et al. (2018) reported that the total polyphenol content in cladode juice could range from approximately 455.65 to 542.70 μg GAE mL^−1^. Similarly, Chahdoura et al. (2024) documented a polyphenol concentration of 58.13 mg GAE g^−1^ of extract, while Moussaoui (2020) recorded a total polyphenol content of 63.54 mg GAE g^−1^ dry matter. These reported values were higher than those obtained in the present study. It is important to emphasize that total polyphenol concentrations are influenced by various factors, including the harvesting location, different developmental stages of the plant, and its age (Santiago‐Saenz et al. 2018).

**TABLE 1:** Total phenolic content (TPC) and total flavonoid content (TFC) of cladode juice of *Opuntia ficus indica*.

	Cladode juice
TPC (mg AGE mL^−1^)	0.389 ± 0.04
TFC (mg QE mL^−1^)	0.187 ± 0.05

Similarly, the quantification of TFC demonstrated that the mean content in the juice, expressed as milligrams of quercetin equivalent per milliliter, was 0.187 ± 0.05 mg QE mL^−1^. The observed flavonoid levels underscore the presence of these secondary metabolites, which are recognized for their diverse bioactive properties, particularly in modulating oxidative stress and contributing to cellular defense systems. In comparison with other studies, Chahdoura et al. (2024) reported that the total flavonoid content in cladodes reached 28.89 mg QE g^−1^ of extract. Similarly, Lahmidi et al. (2023) documented values ranging from 3.77 to 7.55 mg QE 100^−1^ mL of cladode puree, while Moussaoui (2020) recorded a total flavonoid concentration of 18.07 mg QE g^−1^ dry matter. Flavonoid levels fluctuate during cladode maturation, peaking at 1 year of growth before gradually declining (Lahmidi et al. 2023). Flavonoids, a class of phenolic compounds with exceptional antioxidant potential, are regarded as essential dietary components. They are widely recognized for their potent free radical‐scavenging properties (Kumari et al. 2017), cardioprotective effects (Li et al. 2013), anti‐inflammatory and hepatoprotective activities (Chen et al. 2014), as well as their antimicrobial action (Biharee et al. 2020). Despite their promising bioactivities, flavonoids exhibit limited bioavailability, which restricts their efficient utilization by the human body (Wang et al. 2018).

### 3.2 HPLC‐MS Characterization of Phenolic Compounds

**FIGURE 1:**
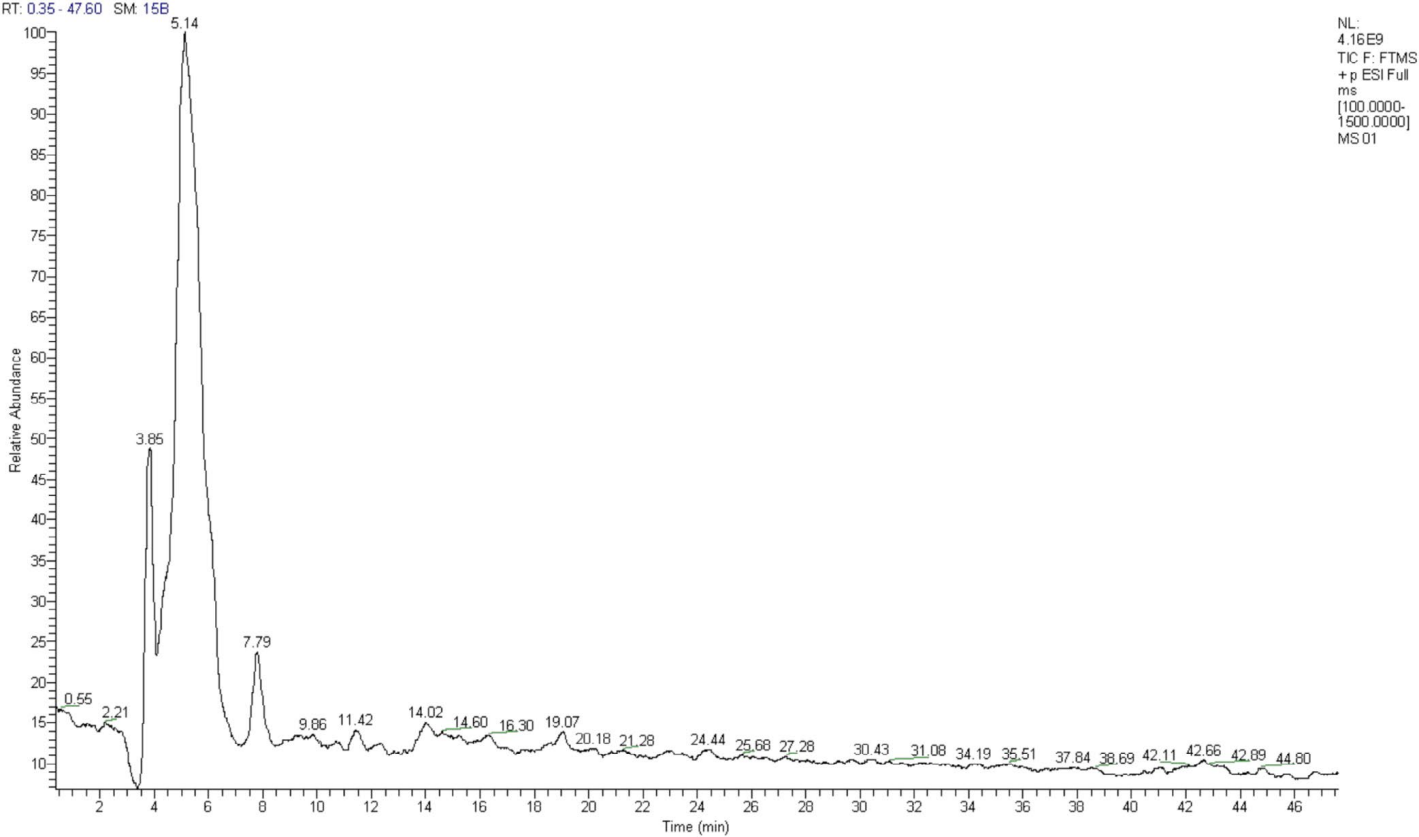
Chromatogram of polyphenols in *Opuntia ficus indica* cladode juice.

The phenolic constituents of cladode juice were elucidated through the comparative analysis of their retention durations and mass spectrometry spectra against established data. Figure 1 presents the identification results of the various peaks observed in the HPLC‐MS chromatogram. Based on the obtained data, four bioactive molecules were identified in the cladode juice: p‐coumaric acid at 4.25 min, piscidic acid at 5.48 min, isorhamnetin‐3‐O‐neohesperoside at 18.95 min, and ellagic acid at 605 min.

p‐Coumaric acid, a phenolic compound belonging to the hydroxycinnamic acid family (Poleto et al. 2024), exhibits high antioxidant activity and plays a crucial role in mitigating diseases associated with oxidative stress. Studies have highlighted its ability to reduce oxidative stress (Chen et al. 2024). Research conducted on mice revealed that p‐coumaric acid protects the liver from damage induced by potassium bromate by alleviating oxidative stress and inflammation (Nivetha et al. 2024).

Piscidic acid, a bioactive compound derived from the cladodes of *OFI*, exhibits significant pharmacological properties, particularly in cholesterol regulation and potential anticancer effects (Ressaissi et al. 2017). This compound demonstrated the ability to inhibit the formation of new blood and lymphatic vessels. Additionally, its antioxidant capacity, comparable to that of carnosic acid, enhances its protective effects on the body (Shibata et al. 2016).

Isorhamnetin‐3‐O‐neohesperoside is a flavonoid glycoside with significant pharmacological properties, particularly in terms of antioxidant and anticancer activities (Du et al. 2016). It is distinguished by its strong antioxidant capacity. This flavonoid also plays a protective role against genetic damage induced by aflatoxin B1 and nifuroxazide (Bouhlel et al. 2010). Moreover, isorhamnetin itself has demonstrated notable anticancer and antifungal potential (Unver 2024; Li et al. 2022).

Administration of isorhamnetin has been shown to reduce lipid accumulation and triglyceride levels, decrease both serum and hepatic lipids, and improve liver lesions (La et al. 2024). Additionally, Yang et al. (2024) observed that isorhamnetin reduces markers of liver damage and oxidative stress, including superoxide dismutase (SOD) and catalase (CAT).

Ellagic acid is a potent polyphenol found in fruits and vegetables, widely recognized for its antioxidant properties and ability to neutralize free radicals (Alfei and Zuccari 2024). However, its bioavailability is limited due to poor water solubility and rapid metabolism (Alfei and Zuccari 2025). It combats aging by reducing oxidative stress, inflammation, and mitochondrial dysfunction. Additionally, it can be metabolized into urolithin, which enhances its bioactivity (Sun 2024).

### 3.3 Effect on Body Weight and Liver Index

The effects of body weight gain and hepatic index in each experimental group are illustrated in Table 2.

**TABLE 2:** Effect of *Opuntia ficus indica* cladode juice on growth parameters in NiSO_4_‐intoxicated rats.

	T	CJ	Ni	CJ + Ni
Weight gain (g)	36.00 ± 2.92	35.60 ± 2.70^a^	1.20 ± 1.79***	22.40 ± 2.30***
Liver index (%)	2.50 ± 0.27	2.57 ± 0.09^a^	3.75 ± 0.05**	3.07 ± 0.19*

*Note:* Mean ± SEM (*n* = 5) is used to express values. T: normal control; JC: cladode juice; Ni: nickel sulphate; JC + Ni: cladode juice and nickel sulphate; JC group: comparison with T group; Ni group: comparison with T group; JC + Ni group: comparison with Ni group.

^a^ Not significant.

***p < 0.001; **p < 0.01; *p < 0.05.

The daily oral administration of CJ at a dosage of 10 mL kg^−1^ body weight for 30 days did not induce any discernible toxic effects in rats. Throughout the study, no mortality or significant clinical abnormalities were found in the group that received treatment, and the animals exhibited normal body weight progression, comparable to that of the control group. Assessments of body mass and hepatic indices revealed no signs of toxicity linked to CJ administration, further supporting the extract's safety profile.

Conversely, NiSO_4_ injections led to a significant reduction in body weight and an increase in the hepatic index, highlighting its hepatotoxic effects. However, oral administration of CJ markedly mitigated these alterations. Intraperitoneal injection of NiSO_4_ resulted in a pronounced decrease in weight gain (*p* < 0.001) and a significant rise in the hepatic index (*p* < 0.01) compared to the control group. These variations indicate liver toxicity and metabolic disturbances.

However, daily administration of CJ for 30 days enhanced growth performance. Treatment with the cladode extract significantly reduced the hepatic index (*p* < 0.05) and improved body weight gain (*p* < 0.001) compared to the NiSO_4_ group, suggesting a protective effect of cladodes against NiSO_4_‐induced toxicity.

The tolerance of CJ, with no signs of toxicity or clinical symptoms, reinforces its potential as a natural hepatoprotective agent. Moreover, the improvement in body weight may reflect not only protection against muscle mass loss but also stimulation of anabolic processes, suggesting that CJ could aid in restoring normal metabolic functions disrupted by toxins such as NiSO_4_.

Several studies have demonstrated that the relative liver weight is a sensitive marker of hepatotoxicity in experimental models of liver injury induced by toxins such as nickel sulfate (NiSO_4_) (Adeyemi and Orekoya 2014; Faoziyat et al. 2014). In line with this, multiple authors have reported that nickel accumulation in the liver is associated with an increase in the hepatic index (Bouhalit and Kechrid 2018; Derbal et al. 2022), further supporting the link between NiSO_4_ exposure and liver dysfunction. While NiSO_4_ exhibits hepatotoxic effects, the cladodes of *OFI* have shown hepatoprotective properties in other toxicity models. Their mode of action, primarily through oxidative stress reduction, suggests a potential to mitigate nickel‐induced liver damage (Brahmi et al. 2011; Makeen et al. 2019). Phenolic compounds in plants are widely recognized for their health benefits, primarily due to their antioxidant properties. Their hydroxyl groups and aromatic structure enable them to neutralize free radicals (Villaño et al. 2007). These effects result from the synergistic action of flavonoids, particularly quercetin, known as the most potent free radical scavenger (Stintzing and Carle 2005; Hfaiedh et al. 2008).

### 3.4 Effect on Enzymatic Biomarkers (ALT, AST, ALP, and GGT)

Table 3 illustrates the effect of *Opuntia ficus indica* cladode juice on the enzymatic activities of alanine aminotransferase (ALT), aspartate aminotransferase (AST), alkaline phosphatase (ALP), and gamma‐glutamyl transferase (GGT).

**TABLE 3:** Effect of *Opuntia ficus indica* cladode juice on ALT, AST, ALP, and GGT enzyme activity in NiSO_4_‐intoxicated rats.

Parameters	Control	CJ	Ni	CJ + Ni
ALT U/L	42.60 ± 0.86	41.01 ± 0.43^a^	65.05 ± 2.61***	49.96 ± 0.38**
AST U/L	99.02 ± 0.08	96.04 ± 0.27^a^	137.94 ± 3.72***	113.80 ± 0.60**
ALP U/L	106.77 ± 2.49	103.40 ± 2.73^a^	266.49 ± 2.62***	220.86 ± 3.11***
GGT U/L	2.16 ± 0.07	1.93 ± 0.03^a^	6.62 ± 0.04***	3.82 ± 0.04**

*Note:* Mean ± SEM (*n* = 5) is used to express values. T: normal control; JC: cladode juice; Ni: nickel sulphate; JC + Ni: cladode juice and nickel sulphate; JC group: comparison with T group; Ni group: comparison with T group; JC + Ni group: comparison with Ni group.

^a^ Not significant.

***p < 0.001; **p < 0.01; *p < 0.05.

#### 3.4.1 Hepatocytolysis (ALT, AST)

The findings demonstrated that nickel exposure (Ni group) elicited a highly significant increase (*p* < 0.001) in serum alanine aminotransferase (ALT) and aspartate aminotransferase (AST) activities, both well‐established biomarkers of hepatocellular injury. These elevations indicate nickel‐induced hepatic impairment. Administration of cladode juice significantly attenuated (*p* < 0.01) these ALT and AST elevations, highlighting its hepatoprotective potential.

The pronounced increase in transaminases (ALT, AST) in the Ni group suggests impaired hepatic function, corroborating findings from previous studies (Lee et al. 2007; Yang et al. 2011; Kuriakose et al. 2017).

Compared to the Ni group, cladode juice administration significantly mitigated these elevations (*p* < 0.01 for ALT and AST) in the JC + Ni group, indicating a beneficial effect on hepatic function. Moreover, cladode juice treatment in the JC group did not induce significant changes in the enzymatic markers assessed, suggesting that its protective effects are specific to nickel‐induced toxicity.

For the diagnosis and ongoing management of liver disorders, the measurement of Hepatocytolysis markers like ALT and AST is essential (Bishnoi et al. 2024). Hepatic dysfunction can be accurately detected biochemically by elevated serum levels of AST and ALT, which are caused by damaged hepatocytes releasing more of these enzymes (Sharma and Shukla 2011; Kharchoufa et al. 2020).

#### 3.4.2 Cholestasis (ALP, GGT)

However, alkaline phosphatase (ALP) and gamma‐glutamyl transferase (GGT) activities, which are associated with cholestatic processes and bile duct dysfunction, were also significantly elevated in the Ni group (*p* < 0.001). Such increases are commonly associated with obstruction or damage to the biliary tract, leading to impaired bile flow and accumulation of bile acids in the liver (Lee et al. 2007; Yang et al. 2011; Kuriakose et al. 2017).

In the JC + Ni group, the addition of cladode juice significantly reduced these increases (*p* < 0.001 for ALP and *p* < 0.01 for GGT) compared to the Nickel (Ni) group, suggesting a beneficial impact on liver function.

Furthermore, the administration of cladode juice in the JC group did not elicit noteworthy alterations in the evaluated cholestasis biomarkers, indicating that its protective properties are particularly tailored to counteract nickel‐induced toxicity.

The potent antioxidant activity of *Opuntia ficus indica* cladodes, attributed to their high content of bioactive phytochemicals such as polyphenols and flavonoids, may underlie their hepatoprotective properties (Slimane et al. 2024). These compounds enhance liver function and confer protection against hepatic injury through their antioxidative and anti‐inflammatory mechanisms (Abbas et al. 2022).

Moreover, GGT levels fluctuate depending on the nature of liver disorders, with their elevation being a highly sensitive marker of hepatic impairment and a valuable tool in clinical investigations (Xing et al. 2022).

### 3.5 Effect on Plasma Direct Bilirubin (DB) and Total Bilirubin (TB) Levels

To evaluate the NiSO_4_ intoxication as well as the potential protective effect of the juice of *Opuntia ficus indica* cladodes (CJ), total (TB), direct (DB), and indirect bilirubin (IB = TB–DB) were measured. The results are indicated in Table 4.

**TABLE 4:** Effect of *Opuntia ficus indica* cladode juice on TB and DB in NiSO_4_‐intoxicated rats.

Parameters	Control	CJ	Ni	CJ + Ni
TB (mg/L)	1.03 ± 0.01	1.07 ± 0.02^a^	3.67 ± 0.13***	2.49 ± 0.13*
DB (mg/L)	0.53 ± 0.03	0.46 ± 0.04^a^	0.89 ± 0.14***	0.62 ± 0.03*
IB (mg/L)	0.51 ± 0.04	0.62 ± 0.06^a^	2.86 ± 0.27***	1.87 ± 0.10*

*Note:* Mean ± SEM (*n* = 5) is used to express values. Ni control group versus normal control group; CJ + Ni versus Ni control group; CJ group versus normal control group.

^a^ Not significant.

***p < 0.001; *p < 0.05.

Exposure to NiSO_4_ resulted in a highly significant increase in total bilirubin levels (3.67 ± 0.13 mg/L; *p* < 0.001) and direct bilirubin levels (0.89 ± 0.14 mg/L; *p* < 0.001) compared to the control group (1.03 ± 0.01 mg/L and 0.53 ± 0.03 mg/L, respectively). The calculation of indirect bilirubin showed a clear increase (2.78 ± 0.27 mg/L against 0.50 ± 0.04 mg/L in the controls), suggesting a mixed liver injury which is characteristically described as “hepatocellular jaundice”. This type of jaundice occurs when the mechanisms of conjugation and bile excretion of bilirubin are both incapacitated, as in cases of toxic hepatitis.

The combined treatment with cladodes juice and NiSO_4_ (CJ + Ni) made it possible to significantly reduce the levels of total bilirubin (2.49 ± 0.13 mg/L; *p* < 0.05), direct bilirubin (0.62 ± 0.03 mg/L; *p <* 0.05), and indirect bilirubin (1.87 ± 0.16 mg/L), thus showing an improvement in liver function. The decrease in these levels indicates an improvement in liver damage and incomplete but recovering hepatobiliary function.

Moreover, the administration of cladode juice alone, without NiSO_4_ exposure, did not lead to significant changes in total, direct, and indirect bilirubin concentrations compared to the healthy control group. The differences observed between the groups did not reach statistical significance (*p* < 0.05). This indicates that, in the absence of toxic stress induced by metals such as nickel, cladode juice does not appear to notably affect bilirubin levels and, therefore, does not disrupt its normal metabolism. These findings suggest that cladode juice has neither hepatotoxic effects nor an impact on bilirubin metabolism in healthy subjects, further supporting its safety when consumed alone.

The administration of NiSO_4_ in rats leads to an elevation in serum bilirubin levels, indicating liver injury (Bouhalit and Kechrid 2018). Similarly, direct bilirubin levels have been positively correlated with the severity of hepatic dysfunction and other conditions related to oxidative stress (Song et al. 2022).

Furthermore, Okaiyeto et al. (2018) reported that elevated serum bilirubin levels are indicative of increased erythrocyte degradation. Additionally, an increase in blood bilirubin levels may result from enhanced tissue destruction or bile duct obstruction (Sabiu et al. 2015).

The therapeutic properties of *OFI* cladodes in improving liver function highlight their potential as a natural remedy for supporting liver health and preventing the progression of metabolic disorders and diabetes‐related complications (Jain et al. 2023). Their hepatoprotective effects are attributed to their anti‐inflammatory and antioxidant properties. Several studies have confirmed their ability to reduce cytokines associated with liver damage, thereby helping to mitigate hepatic injury (Abbas et al. 2022; Mannaa and Abdel‐Wahhab 2016).

### 3.6 Effect on Plasma Total Protein and Glucose Levels

The plasma concentrations of total proteins and glucose in treated and untreated animals are presented in Table 5.

**TABLE 5:** Effect of *Opuntia ficus indica* cladode juice on plasma total protein and glucose levels in NiSO_4_‐intoxicated rats.

Parameters	Control	CJ	Ni	CJ + Ni
Glucose g/L	1.37 ± 0.04	1.17 ± 0.11^a^	2.72 ± 0.07***	1.98 ± 0.06*
T protein mg/L	76.22 ± 0.43	79.86 ± 0.43^a^	55.75 ± 0.17***	69.11 ± 0.54*

*Note:* Mean ± SEM (*n* = 5) is used to express values. Ni control group versus normal control group; CJ + Ni versus Ni control group; CJ group versus normal control group.

^a^ Not significant.

***p < 0.001; *p < 0.05.

Nickel sulfate (NiSO_4_) exposure induced a significant decline in plasma total protein concentrations (*p* < 0.001) while markedly elevating blood glucose levels (*p* < 0.001) compared to the healthy control group. However, cladode juice administration over 30 days exerted a notable protective effect against these biochemical disturbances. This supplementation mitigated the reduction in total protein levels (*p* < 0.05) and effectively restrained the rise in plasma glucose concentrations (*p* < 0.05) observed in the JC + Ni group.

Furthermore, cladode juice administration alone (JC group) resulted in a non‐significant decrease in glucose concentration and a non‐significant increase in total protein concentration compared to the normal control group. The variations observed between these two groups did not reach statistical significance (*p* < 0.05), indicating the absence of a measurable effect of cladode juice on these parameters under normal conditions.

Additionally, in the absence of toxic stress, particularly that induced by nickel exposure, cladode juice does not disrupt the normal metabolism of these substances. These findings further support the notion that cladodes do not exert adverse metabolic effects under non‐toxic conditions and exhibit beneficial modulatory properties, helping to maintain the balance of plasma parameters disrupted by NiSO_4_ exposure. This highlights their safety when consumed under physiological conditions.

Studies have demonstrated that the consumption of *OFI* cladodes leads to a significant reduction in blood glucose levels in rats as well as in healthy individuals following a glucose tolerance test (Butterweck et al. 2010; Proeyen et al. 2012). Research by Avila‐Nava et al. (2014) highlighted a significant increase in plasma antioxidant capacity after the intake of *OFI* extracts, which are rich in phenolic compounds. These findings suggest a promising potential for mitigating oxidative stress and inflammation.

Dahdouh et al. (2014) reported that exposure to NiSO_4_ leads to a significant reduction in serum total protein and albumin levels, indicating liver dysfunction and impaired protein synthesis. Similarly, it has been associated with an increase in blood glucose concentrations, suggesting disruptions in glucose metabolism and a potential risk of insulin resistance (Bouhalit and Kechrid 2018).

### 3.7 Effect on MDA and SOD Activity in NiSO4‐Intoxicated Rats

**FIGURE 2:**
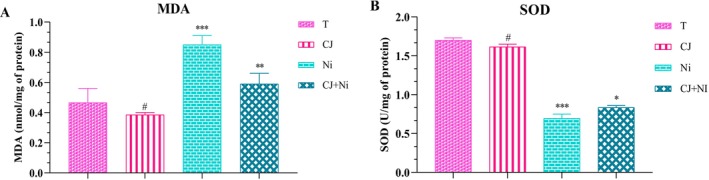
Effect of *Opuntia ficus indica* cladode juice on MDA levels (A) and SOD activity (B) in NiSO_4_‐intoxicated rats. Mean ± SEM (*n* = 5) is used to express values; T: normal control; JC: cladode juice; Ni: nickel sulfate; JC + Ni: cladode juice and nickel sulfate. JC group: comparison with the T group; Ni group: comparison with the T group; JC + Ni group: comparison with the Ni group. ***p < 0.001; **p < 0.01; *p < 0.05; ^#^Non‐significant.

The impact of the effect of OFI cladode juice on lipid peroxidation and SOD activity in treated experimental animals is presented in Figure 2.

Intraperitoneal administration of nickel sulfate (NiSO_4_) in rats triggered pronounced oxidative stress, as evidenced by a significant elevation in hepatic malondialdehyde (MDA) levels (*p* < 0.001), indicating heightened lipid peroxidation and membrane damage driven by reactive oxygen species (ROS). Concurrently, a marked decline in superoxide dismutase (SOD) activity (*p* < 0.001) was observed, suggesting an inhibitory effect of NiSO_4_ on this critical antioxidant enzyme.

Daily administration of *OFI* cladode juice for 30 days in NiSO_4_‐exposed rats led to a significant reduction in MDA levels (*p* < 0.001), reflecting an attenuation of lipid peroxidation due to the antioxidant properties of its bioactive compounds. Furthermore, pre‐exposure supplementation conferred notable protection against oxidative stress, as evidenced by a significant increase in SOD activity (*p* < 0.05) in treated animals. These findings indicate that cladode juice, enriched with antioxidant and anti‐inflammatory agents, exerts a prophylactic effect by preserving cellular redox homeostasis under nickel‐induced oxidative insult.

Moreover, the administration of cladode juice alone in healthy rats led to a slight reduction in hepatic MDA levels, though this decrease did not reach statistical significance (*p* < 0.05), suggesting a mild antioxidant effect in the absence of exogenous oxidative stress. Similarly, no significant alteration in SOD activity was detected, indicating that cladode juice functions as an adaptive modulator, enhancing enzymatic response only under oxidative challenge, such as that induced by NiSO_4_ exposure.

The observed benefits of cladode juice, though more pronounced under induced oxidative stress, may also contribute to maintaining hepatic redox balance in healthy individuals. Preliminary findings suggest that cladodes could enhance baseline antioxidant status by moderately reducing spontaneous lipid peroxidation (Besné‐Eseverri et al. 2023). These effects may stem from the high concentration of phytochemicals such as flavonoids and polyphenols, known for their ability to scavenge reactive oxygen species (ROS) and prevent lipid membrane damage (Zeghbib et al. 2022; Ahmedah 2023).

### 3.8 Effect on GSH, GSH‐Px and Catalase in NiSO4‐Intoxicated Rats

**FIGURE 3:**
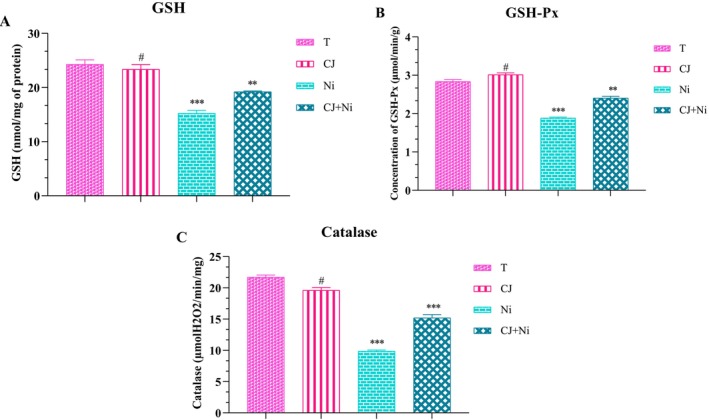
Effect of *Opuntia ficus indica* cladode juice on GSH (A) and GSH‐Px (B) levels, and catalase activity (C) in NiSO_4_‐intoxicated rats. Mean ± SEM (*n* = 5) is used to express values; T: normal control; JC: cladode juice; Ni: nickel sulfate; JC + Ni: cladode juice and nickel sulfate. JC group: comparison with the T group; Ni group: comparison with the T group; JC + Ni group: comparison with the Ni group. ***p < 0.001; **p < 0.01; *p < 0.05; ^#^Non‐significant.

The impact of the juice effect of OFI cladodes on the levels of reduced glutathione (GSH), glutathione peroxidase (GSH‐Px), and catalase (CAT) activity in treated experimental animals is shown in Figure 3.

Intraperitoneal injection of nickel sulfate (NiSO_4_) resulted in a highly significant decrease (*p* < 0.001) in reduced glutathione (GSH), glutathione peroxidase (GSH‐Px), and catalase (CAT) levels in exposed rats compared to the healthy control group. This reduction indicates significant oxidative stress, accompanied by an increase in reactive oxygen species (ROS) production, leading to redox imbalances and increased cellular damage. The decline in GSH reflects an impairment of the primary antioxidant defense system, while the decrease in GSH‐Px and CAT further compromises the hepatic cells' ability to neutralize peroxides and mitigate NiSO_4_‐induced oxidative stress. However, the preventive administration of *OFI* cladode juice for 30 days prior to NiSO_4_ exposure exerted a notable protective effect by alleviating oxidative stress. Compared to untreated intoxicated rats, those receiving cladode juice exhibited significantly higher GSH, GSH‐Px, and CAT levels (*p* < 0.01 to *p* < 0.001), indicating a beneficial modulation of antioxidant defense mechanisms. The increase in CAT levels facilitated the decomposition of hydrogen peroxide (H_2_O_2_) into water and oxygen, thereby reducing its toxic accumulation in hepatic cells. These findings suggest that the bioactive compounds present in *OFI* cladodes enhance the body's antioxidant system and mitigate the deleterious effects of NiSO_4_‐induced oxidative stress, highlighting their protective potential against oxidative agents.

The exclusive administration of *OFI* cladode juice to rats, in the absence of NiSO_4_ exposure, showed no signs of intoxication or notable physiological alterations compared to healthy control rats. Statistical analysis of reduced glutathione (GSH) levels revealed no significant difference (*p* > 0.05) between the group receiving cladode juice alone and the control group, suggesting the absence of toxic effects under normal physiological conditions. Similarly, although an increase in glutathione peroxidase (GSH‐Px) levels was observed in treated rats compared to controls, this elevation did not reach statistical significance (*p* < 0.05), indicating that cladode juice does not exert any harmful effects on cellular antioxidant systems. Conversely, in rats exposed to NiSO_4_, prior administration of cladode juice helped preserve GSH levels and mitigate oxidative stress, suggesting a potential protective role by enhancing intrinsic antioxidant defenses. Regarding catalase (CAT) activity, a slight but non‐significant decrease (*p* < 0.05) was observed in rats receiving cladode juice alone, without indicating any disruption of endogenous antioxidant defense mechanisms. These findings confirm the safety of *OFI* cladode juice and support its potential as a natural, risk‐free supplement capable of modulating oxidative stress induced by toxic agents such as nickel sulfate.

The cladodes of *OFI* contain bioactive compounds, such as polyphenols and flavonoids, which play a key role in modulating antioxidant activity and reducing oxidative damage (Ďuračková 2010; Voronkova et al. 2018). Their consumption can stimulate glutathione peroxidase (GSH‐Px) activity, thereby strengthening cellular antioxidant defenses and helping to mitigate oxidative stress resulting from an imbalance between free radicals and antioxidants. According to Chekkal et al. (2019), young cactus cladodes exert a protective effect against oxidative stress induced by an unbalanced diet, as evidenced by increased GSH‐Px activity and reduced lipid peroxidation. Furthermore, the antioxidant properties of *OFI* cladodes contribute to the preservation of catalase activity in hepatic cells, which is essential for counteracting peroxide accumulation (Rocchetti et al. 2018). Additionally, their anti‐inflammatory effects support liver function by minimizing inflammation, a factor that can compromise catalase activity (Caroline et al. 2024).

### 3.9 Histological Study

**FIGURE 4:**
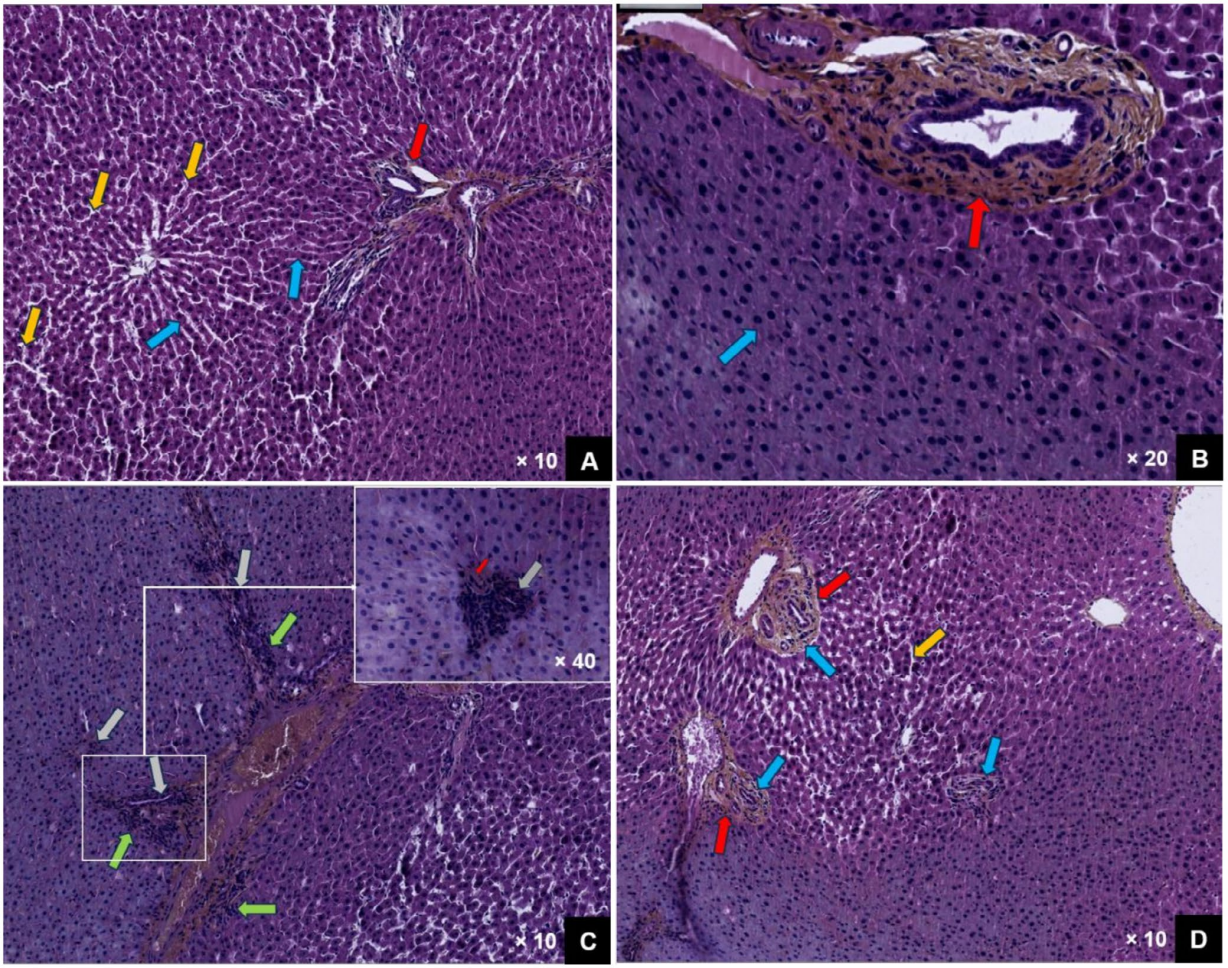
Effect of *OFI* cladode juice on liver histological alterations in NiSO_4_‐intoxicated rats. Histological sections of liver tissue (stained with hematoxylin–eosin) were examined in the normal control group (A), the group treated with cladode juice (B), the group exposed to nickel sulfate (NiSO_4_) (C), and the group receiving cladode juice + NiSO_4_ (D). Observed features include normal sinusoidal dilation (yellow arrow), a portal structure (red arrow), hepatocytic trabeculae (blue arrow), portal inflammation (gray arrow), and interface hepatitis (green arrow).

Figure 4 illustrates the effect of cladode juice on the histological alterations in the liver of rats intoxicated with NiSO_4_.

Histological examination of liver sections reveals marked differences between control rats, NiSO_4_‐exposed rats, and those pretreated with cladode juice (CJ). These observations provide deeper insights into the potential hepatoprotective effects of CJ against oxidative and inflammatory stress induced by nickel exposure, as well as the cellular responses and defense mechanisms involved.

In control rats (Figure 4A), liver sections exhibit hepatic parenchyma with structurally normal characteristics. Hepatocyte trabeculae are regularly aligned, featuring rounded nuclei and showing no signs of lobular necrosis, indicative of hepatic integrity (blue arrow). The portal spaces contain well‐organized portal structures embedded in connective tissue, with a few scattered lymphocytes (red arrow), likely ensuring minimal immune surveillance without triggering an inflammatory response. A mild sinusoidal dilation is observed (yellow arrow), which may be attributed to volumetric overload following increased water intake. These findings confirm the absence of cellular disturbances or functional impairments, providing a morphological reference for the liver under normal physiological conditions.

Administration of cladode juice (Figure 4B) in the absence of NiSO_4_ exposure did not induce any significant alterations in the hepatic parenchyma, suggesting that cladode juice does not interfere with hepatic homeostasis under non‐stressed conditions. Hepatocyte trabeculae remain structurally intact (blue arrow), with no signs of dilation or necrosis, and the portal spaces retain their normal architecture without notable inflammation (red arrow). These findings confirm that cladode juice does not exert hepatotoxic effects on its own but may possess protective and anti‐inflammatory properties in the presence of hepatic injury.

In rats exposed to NiSO_4_, histological examination reveals pronounced hepatic alterations (Figure 4C). Hepatocyte trabeculae exhibit focal sinusoidal dilation, indicative of impaired microcirculation, a phenomenon commonly associated with oxidative stress. This dilation may result from fluid overload due to a nickel‐induced metabolic imbalance, leading to the accumulation of reactive oxygen species in the liver. The portal spaces exhibit dense and chronic inflammatory infiltration (gray arrow), consisting predominantly of lymphocytes with a few polymorphonuclear neutrophils, contributing to mild to moderate interface hepatitis (green arrow). The pro‐inflammatory properties of nickel within the hepatic parenchyma likely account for this portal inflammation and associated interface hepatitis. High‐magnification imaging highlights the severity of the portal inflammatory infiltrate, predominantly composed of lymphocytes and scattered polymorphonuclear cells (gray arrow), nearly obstructing all portal structures, with only a single remaining portal arteriole (red arrow).

Rats pretreated for 30 days with cladode juice before NiSO_4_ exposure exhibit significant protection against nickel‐induced toxicity (Figure 4D). Although mild sinusoidal dilation persists, indicating some degree of microvascular stress, inflammatory infiltration around the portal structures is markedly reduced. The portal space (red arrow) contains only a few scattered lymphocytes within the connective tissue (blue arrow), with no significant inflammatory density, suggesting a mitigation of the excessive immune response triggered by nickel exposure. Additionally, interface hepatitis is completely resolved, with a preserved hepatocyte limiting plate, indicating restoration of hepatocyte membrane integrity and reestablishment of normal trabecular organization.

The observed protective effects can be attributed to the antioxidant and anti‐inflammatory properties of cladode juice, which is rich in bioactive compounds. Previous studies on liver histology have documented nickel‐induced alterations, such as sinusoidal dilation, vacuolization, and hepatocytes exhibiting deformed nuclei (Rao et al. 2009; Fatmi et al. 2018). Furthermore, oxidative stress plays a crucial role in hepatic dysfunction and contributes to the progression of chronic liver diseases (Li et al. 2015). Given their significant involvement in liver failure, antioxidants have been extensively investigated for their potential in preventing and managing both acute and chronic hepatic disorders (Adak 2018). Consequently, numerous studies have demonstrated the beneficial effects of antioxidants, particularly in the prophylaxis and treatment of liver injuries. The prevailing hypothesis suggests that intensive antioxidant intervention may enhance recovery outcomes in cases of hepatic damage (Touiss et al. 2021).

### 3.10 Docking Molecular

**FIGURE 5:**
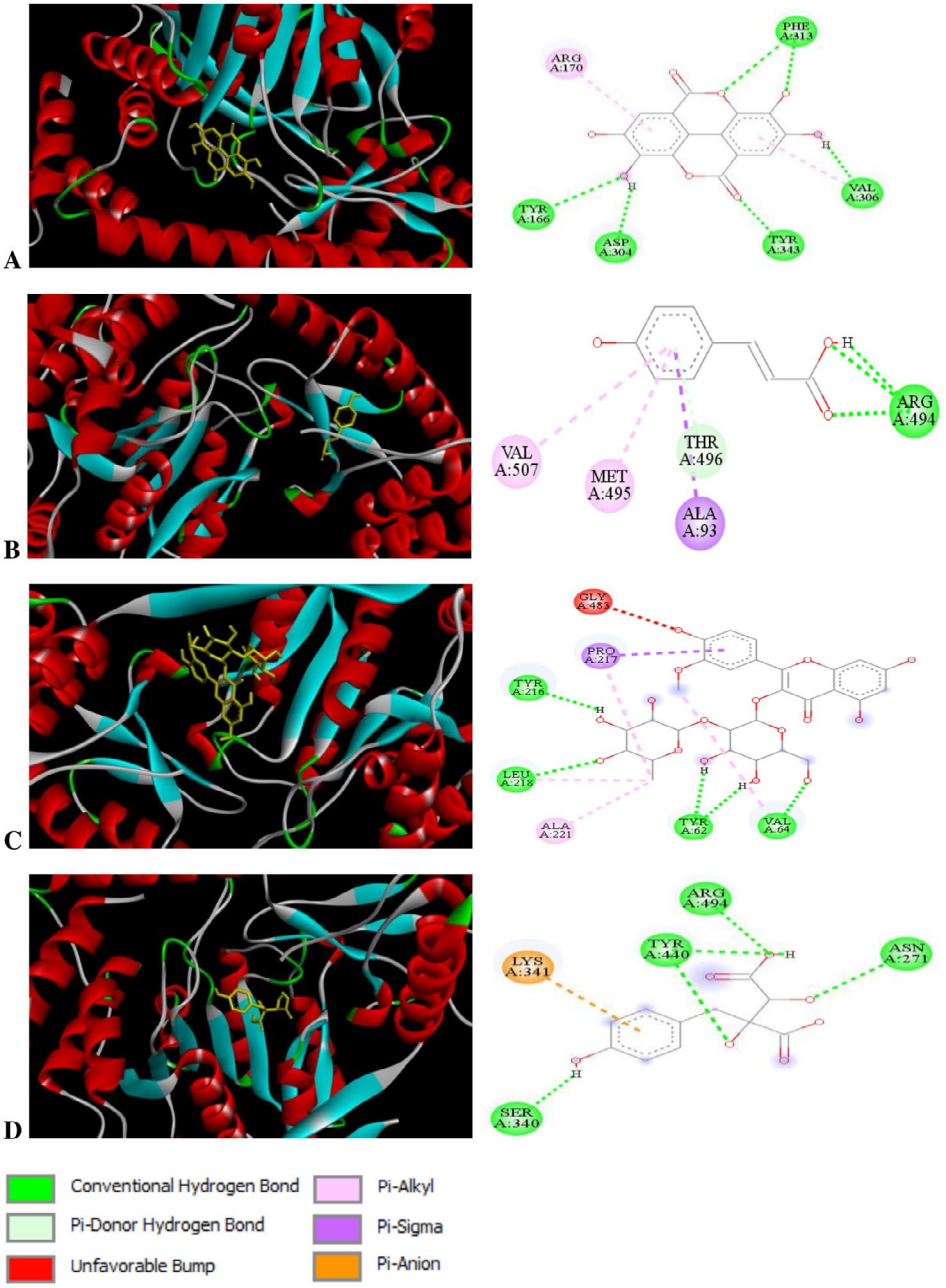
Molecular docking of ellagic acid (A), *p*‐coumaric acid (B), isorhamnetin 3‐O‐neohesperoside (C), and piscidic acid (D) with the target protein.

To achieve our goal, we tested four bioactive compounds against alanine aminotransferase. Molecular docking analysis of these compounds revealed that all of them exhibited highly negative binding energy values. The molecular docking results for the four identified molecules with the target protein are presented in Table 6 and Figure 5.

**TABLE 6:** Interaction scores between bioactive compounds and target protein.

Ligands	Be (Kcal/mol)	Ic (μM)
Ellagic acid	−5.74	61.97
p‐coumaric acid	−6.35	22.27
Isorhamnetin3‐o‐neohesperoside	−2.77	9.36 mM
Piscidic acid	−4.18	867.87

The table shows that p‐coumaric acid with a binding energy of −6.35 Kcal/mol has a stronger interaction than ellagic acid with −5.74 Kcal/mol. Thus, Isorhamnetin 3‐o‐neohesperoside with an Ic of 9.36 mM is less effective than p‐coumaric acid with an Ic of 22.27 μM. Due to its high binding affinity and low inhibitory concentration, p‐coumaric acid appears to be the most promising candidate for therapeutic applications.

The results of the molecular docking study showed that the Ellagic acid compound had five hydrogen bond interactions with the protein residues Val A:306, Tyr A:106, Asp A:304, and Phe A:313. In addition, the compound p‐coumaric acid exhibited an interaction at a hydrogen bond with the protein residue Arg A:494, as well as two pi‐alkyl bonds with the protein residues Val A:507 and Met A:495. The Piscidic acid exhibited a four‐hydrogen bond interaction with the protein residues Ser A:304, Asn A:271, Tyr A:440, and Arg A:494. Isorhamnetin3‐o‐neohesperoside had four hydrogen bonds with the protein residues Tyr A:216, Leu A:218, Tyr A:62, and Val A:64.

After analyzing the molecular docking results of the four compounds, we found that they successfully interacted with and correlated to the binding sites of the selected protein. This strong interaction suggests that these compounds have a high affinity for the target protein, potentially influencing its biological activity. Given their binding properties, it can be inferred that these compounds may exhibit significant antioxidant activity, which could contribute to their potential therapeutic benefits.

It is widely known that bioactive compounds that contain a phenolic ring and a hydroxyl group have superior antioxidant activity (Pripdeevech et al. 2025). Nevertheless, the results of this molecular anchoring research suggest opportunities for the use of cladodes juice as effective antioxidants by reducing oxidative stress.

## 4 Conclusion

This study highlights the hepatoprotective properties of the polyphenols found in *Opuntia ficus indica* cladode juice. Analyses revealed a significant level of phenolic compounds, particularly polyphenols and flavonoids, that contribute to the protective effect on liver function. Assessment of hepatic enzymatic biomarkers and oxidative stress provided significant results, supporting both the safety of the cladode juice and its potential as a protective agent against NiSO_4_‐induced liver damage. Furthermore, histopathological examinations revealed distinct differences between groups, confirming the protective role of the juice against nickel‐induced hepatic lesions. Molecular docking analysis of the four polyphenolic compounds present in the juice demonstrated their interaction with and alignment to the binding sites of target protein, suggesting that these compounds possess notable antioxidant properties.

## Author Contributions

Conceptualization, original draft writing, reviewing, and editing: Sara Razzak, Marouane Aouji, Soufiane El Assri, Chaimae Sabri, Abdulrahman A. Almehizia. Formal analysis, investigations, funding acquisition, reviewing, and editing: Abdessamad Ittorahou, Laila Ibouzinedine, Amir Bouallegue, Esmael M. Alyami. Resources, data validation, data curation, and supervision: Farid Khalouki, Fakhreldeen Dabiellil, Mohammed Bourhia, Anass Haloui, Youness Taboz.

## Disclosure

ARRIVE Guidelines: The experimentation was conducted in accordance with applicable laws, and ARRIVE guidelines.

## Ethics Statement

All experimental procedures were performed in strict accordance with current ethical standards and were approved by the Academic Ethics Committee for Animal Experiments.

## Consent

The authors have nothing to report.

## Conflicts of Interest

The authors declare no conflicts of interest.

## Data Availability

All data generated or analyzed during this study is included in this published article.
