# Correction to “Hepatoprotective Properties of *Opuntia ficus indica* Cladodes Against NiSO_4_‐Induced Oxidative Stress and Liver Damage”

**DOI:** 10.1002/fsn3.71136

**Published:** 2025-10-21

Razzak, S., M. Aouji, S. El Assri, et al. 2025. “Hepatoprotective Properties of *Opuntia ficus indica* Cladodes Against NiSO_4_‐Induced Oxidative Stress and Liver Damage.” *Food Science & Nutrition* 13, no. 10: e71044. https://doi.org/10.1002/fsn3.71044.

The surname of the author, Farid Khalouki, was misspelled in the originally published article. The correct name is Farid Khallouki. The online version has been corrected accordingly.

We apologize for this error.

